# Using Echocardiography and Biomarkers to Determine Prognosis in Peripartum Cardiomyopathy: A Systematic Review

**DOI:** 10.7759/cureus.26130

**Published:** 2022-06-20

**Authors:** Muhammad Sanusi, Elina S Momin, Vijayalakshmi Mannan, Tejasvi Kashyap, Muhammad Ahad Pervaiz, Aqsa Akram, Asma A Khan, Abeer O Elshaikh

**Affiliations:** 1 Internal Medicine, California Institute of Behavioral Neurosciences and Psychology, Fairfield, USA; 2 General Practice, California Institute of Behavioral Neurosciences and Psychology, Fairfield, USA; 3 Urology, California Institute of Behavioral Neurosciences and Psychology, Fairfield, USA; 4 Medicine, California Institute of Behavioral Neurosciences and Psychology, Fairfield, USA

**Keywords:** ppcm, prognosis, biomarkers, echocardiography, cardiomyopathy, pregnancy, peripartum

## Abstract

Peripartum cardiomyopathy (PPCM) is a rare but debilitating form of heart failure that affects pregnant women. Although PPCM has a high rate of complete resolution, some patients often have a progressive disease and develop significant morbidity and mortality. Making an accurate prediction of outcomes and identifying those patients at the highest risk has proven difficult over the years. This study aimed to establish if we can use echocardiographic parameters and biomarkers as reliable indicators of prognosis. A predetermined systematic search strategy was employed in four databases: PubMed, Google Scholar, Science Direct, and Cochrane Library to include articles from the last 15 years (January 2007 to January 2022). Data from 12 studies were synthesized and included in this study. Although no parameter proved consistent in all the studies, echocardiographic parameters, including strain profiles and biomarkers, proved significant in the prognostication of patients with PPCM in the various studies evaluated. Therefore, a holistic approach is still needed in the risk stratification of patients with PPCM. Future studies should evaluate these parameters as well as clinical characteristics in a larger cohort study with a long follow-up period of more than one year in order to potentially develop prognostic score criteria that can be used to accurately identify those patients at the highest risk of developing severe disease or death to allow for timely and targeted therapies to improve outcomes in these patients.

## Introduction and background

One of the leading causes of maternal death in many countries is congenital and acquired heart diseases [1,2]. As recently defined by the working group on peripartum cardiomyopathy (PPCM) of the European Society of Cardiology (ESC), PPCM is the development of heart failure towards the end of pregnancy or in the subsequent months following delivery in a woman with no previously known history of structural heart disease. The left ventricular ejection fraction (LVEF) is usually <45%, but the left ventricle (LV) may be non-dilated [3].

**Figure 1:**
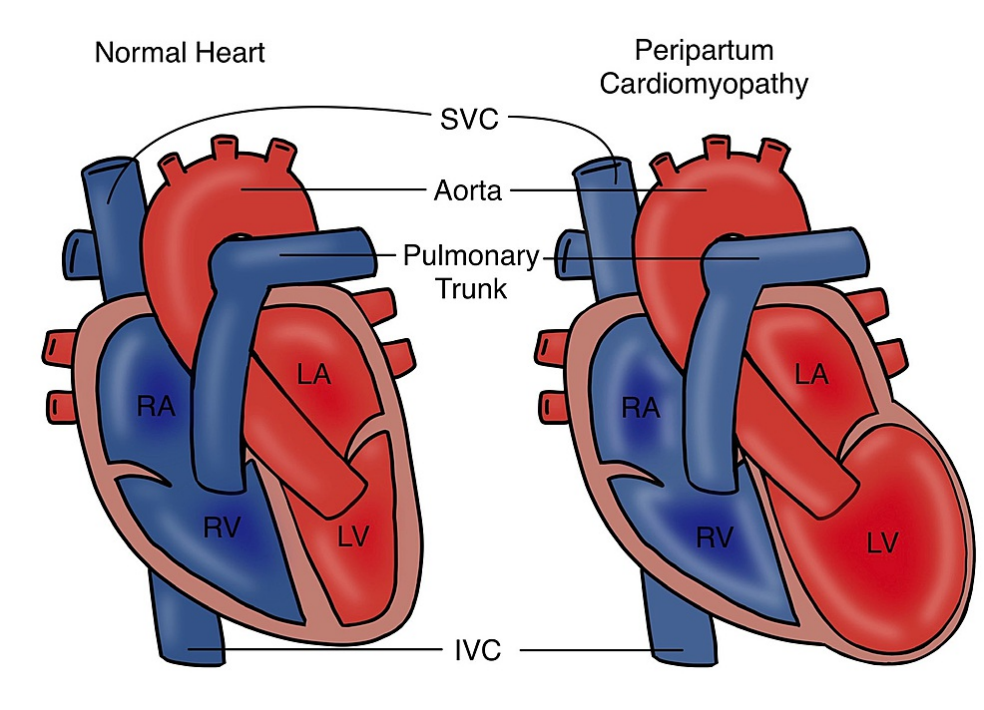
Normal heart vs peripartum cardiomyopathy SVC: superior vena cava, IVC: inferior vena cava, LA: left atrium, LV: left ventricle, RA: right atrium, RV: right ventricle This image is an original illustration by one of the co-authors (Tejasvi Kashyap)

Figure 1 shows a normal heart as compared to a heart with PPCM.

**Figure 2:**
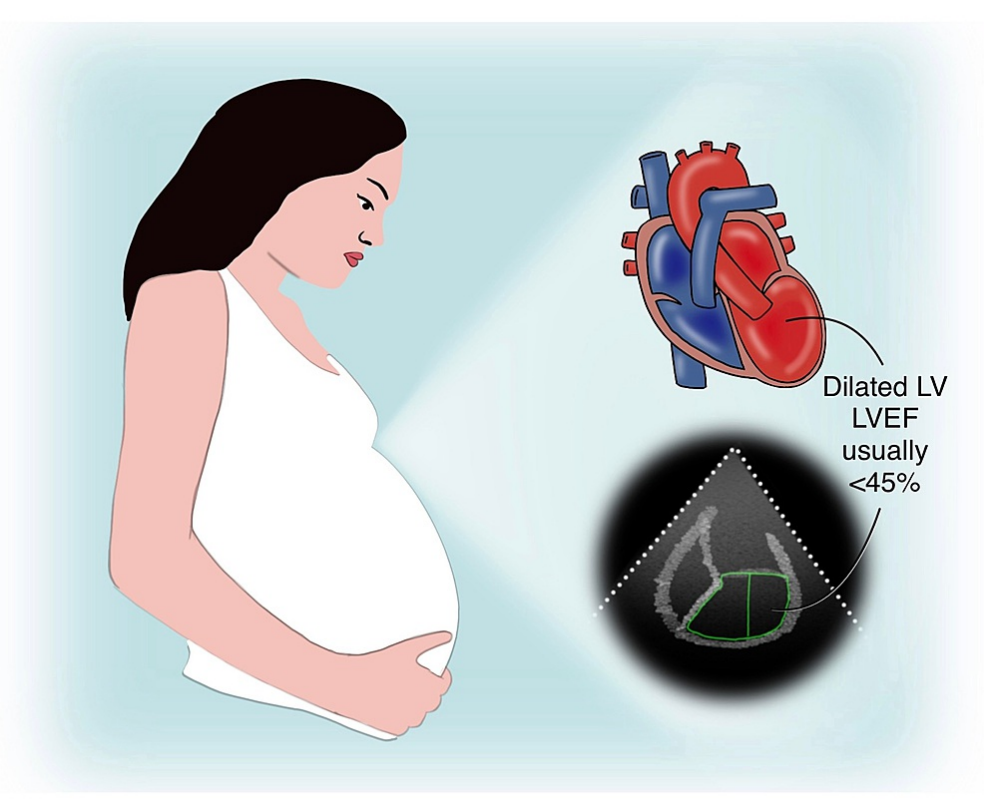
Pregnant woman with PPCM and a dilated heart on echocardiography PPCM: peripartum cardiomyopathy, LV: left ventricle, LVEF: left ventricular ejection fraction, SVC: superior vena cava, IVC: inferior vena cava, LA: left atrium, LV: left ventricle, RA, right atrium, RV: right ventricle This image is an original illustration by one of the co-authors (Tejasvi Kashyap)

Figure 2 shown below demonstrates a pregnant woman with PPCM and a dilated LV on echocardiography.

PPCM is the leading cause of pregnancy-associated heart failure, with a rapid onset, progression, and self-resolution but with a highly significant rate of relapse in subsequent pregnancies [4].

Despite the advances over the years, PPCM remains a poorly understood disease. In particular, data regarding the prediction of outcomes and clinical progression of the disease over time is limited and varies significantly by region [5]. The prospective Investigations of Pregnancy-Associated Cardiomyopathy (IPAC) study, which was done in the United States on patients with PPCM, showed a mortality rate of 4% [6]. On the contrary, a prospective study done in Haiti revealed a mortality rate of 15% and a complete resolution in 28% of the patients [7]. A mortality rate of up to 20% was revealed in another study done by an African collective, albeit they reported a high rate of complete recovery of left ventricular function after two years [8].

Questions commonly arise when patients are diagnosed with a new disease about likely outcomes. Many patients who have been diagnosed with PPCM have admitted to feeling anxious and distraught, struggling with the professional advice to avoid subsequent pregnancy (SSP) and the long-term detrimental effects on their marriage and family affairs [9].

LVEF has long been considered an independent predictor of clinical outcome in patients with PPCM; however, despite some patients having severe systolic dysfunction and markedly reduced LVEF at diagnosis, they tend to recover, which postulates that LVEF alone is insufficient in predicting improvement and the subsequent initiation of premature aggressive therapy such as the use of left ventricular assist devices (LVAD) or transplant [10].

An important biomarker in the diagnosis of heart failure is B-type natriuretic peptide (BNP) and its prohormone peptide, N-terminal B-type natriuretic peptide (NT-proBNP), which are released as a result of cardiac wall stretching [11-13]. When BNP is <100 pg/ml and NT-proBNP is <300 pg/ml, then a diagnosis of PPCM is unlikely [14]. In both symptomatic and asymptomatic patients with heart failure, the NT-proBNP level has been proven to predict prognosis and adverse cardiovascular events [15]. Its prognostic value in patients with PPCM, however, is still under research.

This formed the basis of our research question, "if we can use LVEF as well as other echo parameters and biomarkers as reliable predictors of prognosis in patients with peripartum cardiomyopathy." This systematic review aims to analyze and evaluate those parameters, as the ability to identify early predictors of prognosis can aid in preventing complications, risk stratification, and improving outcomes in this rare but challenging disease.

## Review

Method

This systematic review was designed and carried out using the Preferred Reporting Items for Systematic Reviews and Meta-Analysis (PRISMA) 2020 guidelines [16].

Search Strategy

Four databases - PubMed, Science Direct, Cochrane Library, and Google Scholar - were thoroughly explored electronically. A combination of controlled vocabulary (medical subject headings [MeSH] terms) and advanced search using keywords were used in PubMed and Cochrane Library, while the use of an advanced search strategy using keywords only was employed in Science Direct and Google Scholar to accurately discover all potential articles relevant to our research question. The keywords employed in all databases include peripartum, pregnancy, cardiomyopathy, echocardiography, biomarkers, and prognosis. All databases were last searched on January 31, 2022. A detailed search strategy is provided in the appendix section. Table 1 shows the initial database search results and the results after applying some filters relevant to our study.

**Table 1:** All database search results. Last search January 31, 2022.

	PubMed	Cochrane Library	Science Direct	Google Scholar
Initial search result	988	11	29	5130
Final result filters (2007–2022)	140	8	6	140

Inclusion and Exclusion Criteria

A protocol was developed but not registered with the inclusion criteria of articles published in the English language, human studies, females, free full text only, and articles published in the last 15 years (from January 2007 to January 2022). The selection was also restricted to observational studies and clinical trials. Exclusion criteria included all review articles, editorials, and articles irrelevant to our study. Table 2 shows our full inclusion/exclusion criteria.

**Table 2:** Full inclusion/exclusion criteria.

Inclusion criteria	Exclusion criteria
English language only, female adults, observational studies, randomized clinical trials, non-randomized clinical trials, articles published between January 2007 and January 2022, free full texts only, human studies only	Articles not in English language, review articles, editorials, articles published before 2007, animal studies, grey literature, unavailable free full text, studies not including echocardiography or biomarkers

*Data Selection and Extraction* 

Two reviewers (MS and EM) independently selected and retrieved all potentially relevant articles on Rayyan AI software [17]. Thereafter, the titles, abstracts, and references were thoroughly checked and screened for relevance in the software. All disagreements were resolved by consensus. When disagreements were not resolved, we solicited the aid of a third reviewer (TK).

Risk of Bias Assessment

Assessment of quality was done by two independent researchers (MS and TK). Any disagreements were resolved by consensus or with the aid of a third reviewer (EM). We used the Joanna Briggs Institute critical appraisal tools to critically appraise the quality of the studies, requiring a predetermined quality appraisal cut-off of 70% for the studies to be eligible for inclusion in our study.

Results 

**Figure 3:**
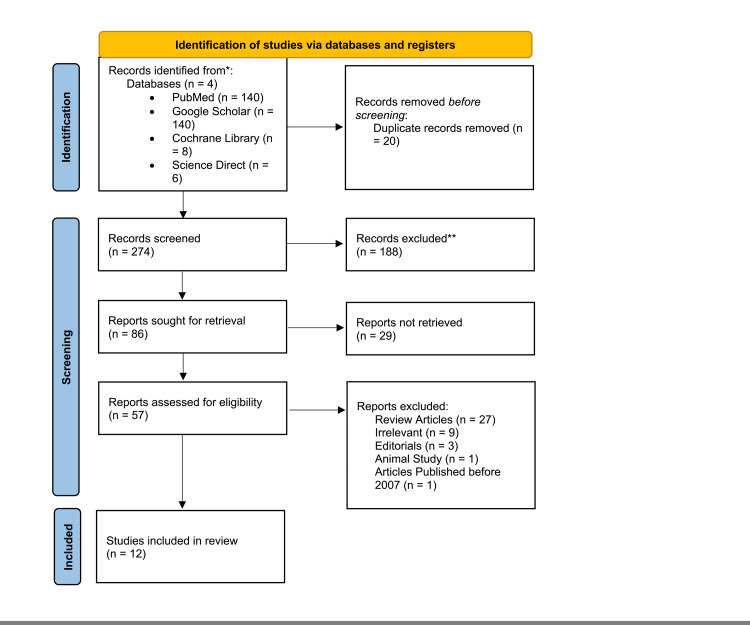
Prisma flow diagram

We identified a total of 294 articles after applying our various search strategies across the four databases. We identified 140 articles from PubMed, 140 articles from Google Scholar, a total of eight articles from the Cochrane Library, and six articles from Science Direct. A total of 20 duplicates were found and removed manually. The remaining 274 articles were thoroughly searched and evaluated for relevance using the titles and abstracts. A total of 188 articles were found to be irrelevant and were removed. Subsequently, 86 articles were sought for the retrieval of full texts. A total of 29 articles were not retrieved and subsequently removed. Fifty-seven full texts were retrieved to be assessed further for relevance and full eligibility. A further 41 articles were removed as they did not fulfill our inclusion/exclusion criteria. Sixteen articles were assessed for quality appraisal, and a further four articles were removed as they were deemed to be of lower quality (<70%) as per our criteria. A total of 12 articles were included in our study; eight were prospective cohort studies, three were retrospective cohort studies, and one was a case series. A complete PRISMA flow diagram is provided below in Figure 3.

Discussion

For a long time, accurately identifying patients with PPCM at the highest risk of significant morbidity and mortality has proven problematic. This systematic review is focused on identifying possible parameters that place patients at the highest spectrum of illness. To the best of our knowledge, this is the first review to evaluate both echocardiographic and biological markers in the prognostication of patients with PPCM. Mortality ranged from none to a high of 18% and up to 24% in the Karaye et al. and Biteker et al. studies, respectively [18,19]. Deaths were reportedly commonly due to the progression of the disease process or sudden cardiac death. Predictors of mortality in the Biteker et al. study were lower ejection fraction and higher left ventricular end-systolic diameter. It was also discovered that all patients that died had a LVEF <40% at their last visit [19]. Patients who died were also noted to have significantly higher levels of BNP at six months [19]. In the Karaye et al. study, patients with tachycardia, hypotension, LVEF <25, and maternal age <21 were two times at risk of death [18]. Although obesity was reported as an independent risk factor for death in the Karaye et al. study, it was found to be a good prognostic factor in the univariate analysis of the McNamara et al. study [6]. However, multivariate regression analysis failed to show this association.

It has also been a challenge to determine when in the disease process to stratify patients as recovered or non-recovered. In one study, some patients (about 30%) recovered within the first six months, and a significant amount recovered beyond that timeline. Among the patients who recovered, about 60% of them achieved recovery beyond the first one-year postpartum. Some patients also developed late deterioration in left ventricular function after one year postpartum after achieving initial recovery [19]. This suggests that the follow-up period should be longer than one year postpartum to accurately determine recovery or non-recovery in patients with PPCM.

Echocardiography Assessment

Exactly half of the studies concluded that LVEF at presentation is a reliable marker for predicting worse outcomes in patients with PPCM [6,18-22]. In contrast, however, patients with a higher median baseline LVEF recovered less than those with lower LVEF at presentation in another study (improved median = 23 vs 32 in the non-improved, p-value = 0.0084) [23]. A baseline LVEF was not statistically significant in predicting a subsequent recovery in a multivariate analysis done by Kiran et al. (P-value = 0.09) [24]. There was no consistent cut-off value reported for LVEF in predicting a worse prognosis. Values ranged from <34% to as low as 25% in one study [18,20]. Another study revealed LVEF <30% is associated with a lack of recovery in univariate analysis, however, multivariate analysis failed to illustrate a statistical significance (Beta = 0.04, SE = 0.14, P-value = 0.79) [6]. However, it was discovered in the same study that no patients with both baseline LVEF <30% and LV end-diastolic diameter (LVEDD) ≥6 cm recovered. In contrast, almost all patients (about 91%) with LVEF ≥30% and LVEDD <6 cm recovered (P-value = <0.00001) [6]. Event-free survival was also far worse for patients that had baseline LVEF <30% compared with those with LVEF ≥30% (one-year event-free survival rate, 82% vs 99%, P-value = 0.004) [6]. Some of the studies failed to report a cut-off value but reported that lower LVEF at presentation was associated with worse outcomes. In another study, baseline LVEF and other parameters failed to show a correlation between patients who improved and those who did not (improved median = 29±9, non-improved median = 28±10, p-value = 0.9) [25].

Other echocardiographic parameters useful in evaluating patients with PPCM include left ventricular end-systolic diameter (LVESD), left ventricular end-diastolic diameter (LVEDD), left ventricular fractional shortening (LVFS), left atrial volume index (LAVi), and right ventricle fractional area change (RVFAC). It was reported that LVEDD >60 to 64 mm, LVFS <16%, LAVi > 29.6 ml/m2, and RVFAC <31.4% were associated with worse outcomes in various studies [6,20,24]. A study done by Prasad et al. revealed marked baseline differences in echo parameters done between improved versus nonimproved (LVEF: 28.7% vs 22.4%, LVEDD: 5.6 cm vs 6.06 cm, LVFS: 17.5% vs 13.4%) and were all statistically significant (P-value < 0.01).

Left ventricular strain profiles at presentation are also an important factor to be considered when evaluating patients with PPCM. Notably, Global Longitudinal Strain (GLS) >10.6% and Global Circumferential Strain (GCS) >10.1% (GLS OR = 1.97 95% CI = 1.42-2.47, P ≤ 0.01, GCS OR = 1.44 95%, CI = 1.20-1.73, P ≤ 0.01) have been found to be associated with worse outcomes [26]. Prediction of clinical outcomes also improved significantly with the addition of GLS and GCS to LVEF, providing a more robust incremental value over the use of LVEF alone. Although there was no single predictor of worse outcomes consistent in all the studies, LVEF at presentation proved to be the most reliable across the studies. The further addition of strain profiles to LVEF proved to have a significant incremental value in predicting recovery.

Biomarkers Assessment

Hormones: Both BNP and NT-proBNP have long been used in the evaluation of patients with heart failure [27]. In recent times, both hormones have demonstrated their importance in the diagnosis and prognosis stratification of patients with PPCM. As earlier reported, in patients with BNP <100 pg/ml and/or NT-proBNP <300 pg/ml, the diagnosis of PPCM is highly unlikely [14]. Their prognostic value also cannot be overemphasized, as demonstrated by the Weiping et al. and Hoevelmann et al. studies. Patients with BNP of >1860 pg/ml (HR 4.74, 95% CI 2.11-10.63, P ≤ 0.001) at baseline have been associated with worse outcomes and less likelihood of recovery [20]. Similarly, multiple regression analysis found that PPCM patients with NT-proBNP of ≥900 pg/ml had persistent LV systolic dysfunction (LVEF <50%; OR 0.20, 95% CI 0.04-0.89, P-value = 0.035) at 12 months. Patients with NT-proBNP ≥900 pg/ml also had poor recovery of LV dimension (LVEDD <55 mm; OR 0.22, 95% CI 0.05-0.95, p-value = 0.043) over the same period [28]. In contrast, in the study by Biteker et al., BNP was not found to be predictive of recovery of left ventricular systolic function as baseline BNP levels were similar among early recovery, delayed recovery, and non-recovery groups [19]. Another study by Forster et al. reported higher levels of baseline NT-proBNP among those that improved compared to those that did not [23]. There was also a strong correlation of NT-proBNP over time with NYHA (New York Heart Association) class, markers of inflammation (oxidized low-density lipoprotein, interferon-gamma), and prolactin, which suggests that NT-proBNP can be used to detect ongoing inflammation or progression of the disease [23]. However, these results are not conclusive and require further evaluation in a study with a larger sample size.

Prolactin, especially the 16-kDa prototype, a major mediator that has been implicated in the pathogenesis of PPCM [29], failed to demonstrate a clear prognostic value at baseline evaluation; however, a marked baseline difference was noted between patients with (PPCM median = 24.7 ng/ml, range 9.6-66.6 vs controls median of 7.40 ng/ml, range 2.85-18.95, P 0.0001), which indicates its usefulness as a marker for diagnosis [23]. There was, however, a significant reduction of prolactin levels at six months in patients who improved as compared to their baseline levels (19.6 ng/ml, range 7.8-43.5, median D 9.2 ng/ml, P = 0.0068) [23]. This suggests that failure of prolactin levels to decrease is associated with a lack of recovery and persistent elevations in prolactin levels at six months are associated with ongoing pathogenesis of the disease process. However, the sample size in this study is relatively small, and the results are in no way definitive.

Inflammatory Markers/Others

Since inflammation and oxidative stress have been proposed as the mediators of pathogenesis in patients with PPCM, many markers of inflammation and oxidative stress have been studied, especially cathepsin D-16-kDa prolactin cascade, which seems to have a central role in decreasing cardiomyocyte function [29]. In the study by Olaf et al., persistent elevation of oxidized low-density lipoprotein (ox-LDL) and interferon-gamma (INF-g) has been associated with a lack of recovery. In another study by Ekizler et al., higher levels of inflammatory markers such as c-reactive protein (CRP), white blood cell (WBC), monocyte and monocyte/HDL cholesterol ratio (MHR). In particular, MHR <9.73 predicted persistent systolic dysfunction with a sensitivity of 89% and specificity of 79% [21]. Higher levels of MHR in patients with PPCM have been proposed to have a pro-inflammatory and pro-oxidant effect on cardiomyocyte function [21].

In another study by Damp et al., they proposed that the pathogenesis of PPCM has at least some components of vascular endothelial dysfunction. Relaxin is a peptide that has a systemic vasodilatory effect. Relaxin-2 has shown some cardioprotective role and its presence, particularly when obtained within the first 11 days postpartum, was associated with a greater likelihood of recovery and less left ventricular remodeling in patients with PPCM. Rapid recovery of systolic function has also been associated with higher levels of Relaxin-2 [30]. In contrast, soluble Fms-like tyrosine kinase 1 (sFlt1) and antiangiogenic factors released from the placenta during the peripartum period have been implicated in the pathogenesis of PPCM and higher levels are associated with the progression of the disease process, less likelihood of recovery, and a higher mortality rate [30]. Table 3 summarizes the prognosis of PPCM patients in the 12 included studies.

**Table 3:** Baseline characteristics of included studies and markers of prognosis in patients with PPCM LVEF: left ventricular ejection fraction; BNP: B-type natriuretic peptide; LVFS: left ventricular fractional shortening; LVEDD: left ventricular end diastolic diameter; oxLDL: oxidized low-density lipoprotein; NT-proBNP: N-terminal pro-B-type natriuretic peptide; IFN-g: interferon-gamma; NYHA: New York Heart Association; GLS: Global Longitudinal Strain; GCS: Global Circumferential Strain; HDL-C: high-density lipoprotein C; CRP: C-reactive protein; WBC: white blood cell; MHR: monocyte to high-density lipoprotein ratio; sFlt1: soluble Fms-like tyrosine kinase 1; BMI: body mass index; Uni: univariate analysis; LVESD: left ventricular end-systolic diameter; LAVi: left atrial volume index; RVFAC: right ventricle fractional area change.

First author/year	Study type	Location	Number of patients	Mean age (years)	Mean follow-up (months)	Definition of recovery	Baseline LVEF (%)	Baseline LVEF % (recovered)	Baseline LVEF % (non-recovered)	Predictors of poor prognosis	Predictors of recovery	Mortality
McNamara et al. [6]	Prospective cohort	United States of America	100	30±6	12	LVEF ≥ 50%	35±10			(1) LVEF≤30% (uni only); (2) LVEDD ≥ 6 cm; (3) BMI (uni only); (4) black race; (5) days postpartum to presentation (uni only)		4 (4%)
Karaye et al. [18]	Prospective cohort	Nigeria	244	28.9±7.2	17	LVEF ≥ 55%	30.1±7.4			(1) LVEF<25%; (2) hypotension; (3) tachycardia; (4) maternal age <20	(1) Beta-blocker therapy; (2) obesity	45 (19%)
Biteker et al. [19]	Prospective cohort	Turkey	42	27±5.2	38.9±14.7	LVEF > 50%	22.1±6.1	(1) Early = 30.7 ± 3.2 2) Delayed= 29.3±4.7	25.7±6.5	(1) LVEF; (2) LVESD		10 (24%)
Li et al. [20]	Retrospective cohort	China	71	28±6	43±33	LVEF ≥ 45%	36.1±6.6	39.5±4.4	31.6±6.3	(1) LVEF<34%; (2) BNP > 1860 pg/ml; (3) LVFS <16 cm (uni only); (4) LVEDD >64 mm (uni only)		0
Ekizler and Cay [21]	Retrospective cohort	Turkey	64	29.2±6	72.1±5.5	LVEF > 45		36	29	(1) LVEF; (2) HDL-C; (3) CRP; (4) WBC; (5) monocyte; (6) MHR <9.73		5 (8%)
Prasad et al. [22]	Case series	India	16	25.25	12	LVEF ≥ 50%	22.4±1.51	28.7	22.4	(1) LVEF; (2) LVEDD; (3) LVFS		1 (6%)
Forster et al. [23]	Prospective cohort	South Africa	43	30	6		29.5	23	32	(1) oxLDL; (2) NT-proBNP; (3) IFN-g; (4) prolactin at 6 months		3 (7%)
Kiran et al. [24]	Prospective cohort	India	43	25.4	6	LVEF > 55%	34.7			(1) LVEF (uni only); (2) LAVi > 29.6 ml/m^2^; (3) RVFAC<31.4%		2 (5%)
Pillarisetti et al, [25]	Retrospective cohort	United States of America	100	30±6.5	35±21	LVEF > 50%	28±9.9	29±9	28±10		(1) Caucasian/Hispanic race; (2) postpartum diagnosis	11 (11%)
Sugahara et al. [26]	Prospective cohort	United States of America	100	31	12	LVEF > 50%	35.6			(1) GLS>10.6%; (2) GCS>10.1%		2 (2%)
Hoevelmann et al. [28]	Prospective cohort	South Africa	35	30±5.9	12	(1) LVEDD <55 mm; (2) LVEF ≥50%	31	33	28	(1) NT-proBNP ≥900 pg/ml; (2) NYHA III or IV 3) Heart rate 4) Sinus tachycardia		
Damp et al. [30]	Prospective cohort	United States of America	100	30±6	12		35±9			sFlt1	Relaxin-2	

Limitations

This study is not without limitations. As PPCM is a rare disease, most of the data available on clinical characteristics and outcomes are diverse and heterogeneous. Likewise, the sample size of most of the studies was relatively small with short follow-up periods. This might have affected the power of the studies to detect a statistically significant difference in some of the parameters evaluated. This study also did not evaluate other investigations like electrocardiography and cardiac magnetic resonance imaging that might be useful in prognostication. It is a limitation that our study only included articles published in the English language only, free full texts only, and the exclusion of all review articles. This might have removed some potentially relevant articles.

## Conclusions

PPCM is a rare but debilitating disease that affects pregnant women. Reliable markers of prognosis have been difficult to identify over the years. Our study evaluated both echocardiographic parameters and blood biomarkers as independent markers of prognosis. We found that many parameters have some prognostic value, but none proved consistent enough to be used alone, while some parameters are novel and have not been evaluated in enough studies to be used independently for predicting prognosis. Therefore, we suggest that a holistic approach should still be employed using clinical characteristics and echocardiographic parameters including strain profiles and biomarkers in order to accurately identify patients at high risk of death or lack of LV recovery to allow for timely and/or aggressive intervention to improve the outcome in these patients. Further research should focus on evaluating the parameters evaluated in this study as well as other parameters not evaluated in this study, e.g., ECG and cardiac MRI in a study with a larger sample size and a long follow-up period of more than one year so that reliable prognostic score criteria can be developed.

## Appendices

Search strategy

PubMed

The final search strategy on PubMed: (“cardiomyopathie”[All Fields] OR “cardiomyopathies”[MeSH Terms] OR “cardiomyopathies”[All Fields] OR “cardiomyopathy”[All Fields] OR (“heart failure”[MeSH Terms] OR (“heart”[All Fields] AND “failure”[All Fields]) OR “heart failure”[All Fields]) OR (“dilatable”[All Fields] OR “dilatated”[All Fields] OR “dilatating”[All Fields] OR “dilatation”[MeSH Terms] OR “dilatation”[All Fields] OR “dilatations”[All Fields] OR “dilate”[All Fields] OR “dilation”[All Fields] OR “dilations”[All Fields] OR “dilatative”[All Fields] OR “dilatator”[All Fields] OR “dilatators”[All Fields] OR “dilated”[All Fields] OR “dilates”[All Fields] OR “dilating”[All Fields] OR “dilator”[All Fields] OR “dilators”[All Fields]) OR “PPCM”[All Fields] OR (“heart failure”[MeSH Terms] OR (“heart”[All Fields] AND “failure”[All Fields]) OR “heart failure”[All Fields] OR (“cardiac”[All Fields] AND “failure”[All Fields]) OR “cardiac failure”[All Fields]) OR (“cardiomyopathy, dilated/blood”[MeSH Terms] OR “cardiomyopathy, dilated/diagnosis”[MeSH Terms] OR “cardiomyopathy, dilated/diagnostic imaging”[MeSH Terms] OR “cardiomyopathy, dilated/mortality”[MeSH Terms])) AND (“peripartum period”[MeSH Terms] OR (“peripartum”[All Fields] AND “period”[All Fields]) OR “peripartum period”[All Fields] OR “peripartum”[All Fields] OR (“Pregnancy”[MeSH Terms] OR “Pregnancy”[All Fields] OR “pregnancies”[All Fields] OR “pregnancy s”[All Fields]) OR (“postpartum period”[MeSH Terms] OR (“postpartum”[All Fields] AND “period”[All Fields]) OR “postpartum period”[All Fields] OR “puerperium”[All Fields]) OR (“postpartum period”[MeSH Terms] OR (“postpartum”[All Fields] AND “period”[All Fields]) OR “postpartum period”[All Fields] OR “postpartum”[All Fields]) OR “Pregnancy”[MeSH Terms]) AND (“Prognosis”[MeSH Terms] OR “Prognosis”[All Fields] OR “prognoses”[All Fields] OR (“outcome”[All Fields] OR “outcomes”[All Fields]) OR (“mortality”[MeSH Terms] OR “mortality”[All Fields] OR “mortalities”[All Fields] OR “mortality”[MeSH Subheading]) OR (“recoveries”[All Fields] OR “recovery”[All Fields]) OR (“epidemiology”[MeSH Subheading] OR “epidemiology”[All Fields] OR “morbidity”[All Fields] OR “morbidity”[MeSH Terms] OR “morbid”[All Fields] OR “morbidities”[All Fields] OR “morbids”[All Fields]) OR “Prognosis”[MeSH Terms]) AND (“biomarker s”[All Fields] OR “Biomarkers”[MeSH Terms] OR “Biomarkers”[All Fields] OR “biomarker”[All Fields] OR (“troponin”[MeSH Terms] OR “troponin”[All Fields] OR “troponins”[All Fields] OR “troponine”[All Fields]) OR “BNP”[All Fields] OR (“natriuretic peptides”[MeSH Terms] OR (“natriuretic”[All Fields] AND “peptides”[All Fields]) OR “natriuretic peptides”[All Fields] OR (“natriuretic”[All Fields] AND “peptide”[All Fields]) OR “natriuretic peptide”[All Fields]) OR (“molecule”[All Fields] OR “molecule s”[All Fields] OR “molecules”[All Fields]) OR (“chemical”[All Fields] OR “chemical s”[All Fields] OR “chemically”[All Fields] OR “chemicals”[All Fields]) OR “Biomarkers”[MeSH Terms] OR (“echocardiographies”[All Fields] OR “Echocardiography”[MeSH Terms] OR “Echocardiography”[All Fields] OR (“echo”[Journal] OR “echo”[All Fields]) OR “TEE”[All Fields] OR “TTE”[All Fields] OR (“Echocardiography”[MeSH Terms] OR “Echocardiography”[All Fields] OR “echocardiogram”[All Fields] OR “echocardiograms”[All Fields]) OR “Echocardiography”[MeSH Terms])) AND (((((((((“cardiomyopathie”[All Fields] OR “cardiomyopathies”[MeSH Terms] OR “cardiomyopathies”[All Fields] OR “cardiomyopathy”[All Fields] OR (“heart failure”[MeSH Terms] OR (“heart”[All Fields] AND “failure”[All Fields]) OR “heart failure”[All Fields]) OR (“dilatable”[All Fields] OR “dilatated”[All Fields] OR “dilatating”[All Fields] OR “dilatation”[MeSH Terms] OR “dilatation”[All Fields] OR “dilatations”[All Fields] OR “dilate”[All Fields] OR “dilation”[All Fields] OR “dilations”[All Fields] OR “dilatative”[All Fields] OR “dilatator”[All Fields] OR “dilatators”[All Fields] OR “dilated”[All Fields] OR “dilates”[All Fields] OR “dilating”[All Fields] OR “dilator”[All Fields] OR “dilators”[All Fields]) OR “PPCM”[All Fields] OR (“heart failure”[MeSH Terms] OR (“heart”[All Fields] AND “failure”[All Fields]) OR “heart failure”[All Fields] OR (“cardiac”[All Fields] AND “failure”[All Fields]) OR “cardiac failure”[All Fields])) AND (“peripartum period”[MeSH Terms] OR (“peripartum”[All Fields] AND “period”[All Fields]) OR “peripartum period”[All Fields] OR “peripartum”[All Fields])) OR (“Pregnancy”[MeSH Terms] OR “Pregnancy”[All Fields] OR “pregnancies”[All Fields] OR “pregnancy s”[All Fields]) OR (“postpartum period”[MeSH Terms] OR (“postpartum”[All Fields] AND “period”[All Fields]) OR “postpartum period”[All Fields] OR “puerperium”[All Fields]) OR (“postpartum period”[MeSH Terms] OR (“postpartum”[All Fields] AND “period”[All Fields]) OR “postpartum period”[All Fields] OR “postpartum”[All Fields])) AND (“Prognosis”[MeSH Terms] OR “Prognosis”[All Fields] OR “prognoses”[All Fields])) OR (“outcome”[All Fields] OR “outcomes”[All Fields]) OR (“mortality”[MeSH Terms] OR “mortality”[All Fields] OR “mortalities”[All Fields] OR “mortality”[MeSH Subheading]) OR (“recoveries”[All Fields] OR “recovery”[All Fields]) OR (“epidemiology”[MeSH Subheading] OR “epidemiology”[All Fields] OR “morbidity”[All Fields] OR “morbidity”[MeSH Terms] OR “morbid”[All Fields] OR “morbidities”[All Fields] OR “morbids”[All Fields])) AND (“biomarker s”[All Fields] OR “Biomarkers”[MeSH Terms] OR “Biomarkers”[All Fields] OR “biomarker”[All Fields])) OR (“troponin”[MeSH Terms] OR “troponin”[All Fields] OR “troponins”[All Fields] OR “troponine”[All Fields]) OR “BNP”[All Fields] OR (“natriuretic peptides”[MeSH Terms] OR (“natriuretic”[All Fields] AND “peptides”[All Fields]) OR “natriuretic peptides”[All Fields] OR (“natriuretic”[All Fields] AND “peptide”[All Fields]) OR “natriuretic peptide”[All Fields]) OR (“molecule”[All Fields] OR “molecule s”[All Fields] OR “molecules”[All Fields]) OR (“chemical”[All Fields] OR “chemical s”[All Fields] OR “chemically”[All Fields] OR “chemicals”[All Fields])) AND (“echocardiographies”[All Fields] OR “Echocardiography”[MeSH Terms] OR “Echocardiography”[All Fields])) OR (“echo”[Journal] OR “echo”[All Fields]) OR “TEE”[All Fields] OR “TTE”[All Fields] OR (“Echocardiography”[MeSH Terms] OR “Echocardiography”[All Fields] OR “echocardiogram”[All Fields] OR “echocardiograms”[All Fields])).

Table 4 shown below shows our full PubMed search strategy.

**Table 4:** PubMed search strategy (last search January 29, 2022)

MeSH Search	Cardiomyopathy #1 Cardiomyopathy OR heart failure OR dilated OR PPCM OR Cardiac failure OR(“Cardiomyopathy, Dilated/blood”[Mesh] OR “Cardiomyopathy, Dilated/diagnosis”[Mesh] OR “Cardiomyopathy, Dilated/diagnostic imaging”[Mesh] OR “Cardiomyopathy, Dilated/mortality”[Mesh])	Pregnancy #2 Peripartum OR pregnancy OR puerperium OR postpartum OR “Pregnancy”[Mesh]	Prognosis #3 Prognosis OR outcome OR mortality OR recovery OR morbidity OR “Prognosis”[Mesh]	Biomarkers #4 Biomarkers OR Troponin OR BNP OR natriuretic peptide OR molecules OR chemicals OR “Biomarkers”[Mesh]	Echocardiography #5 Echocardiography OR Echo OR TEE OR TTE OR Echocardiogram OR “Echocardiography”[Mesh]	#1 AND #2 AND #3 AND (#4 OR #5)	Field Search (Regular Keywords)	Combined mesh + Field Search	Combined Mesh + Field Search with Filters (Free full text, Humans, English, Female, Adult: 19+ years, from 2007 to 2022)
Articles	548,655	1,091,827	6,482,720	4,099,385	261,222	1,499	261,223	988	140

Cochrane Library

Table 5 shown below shows our full search strategy on Cochrane Library.

**Table 5:** Cochrane library search strategy (last search January 31, 2022)

ID	Search	Hits
#1	MeSH descriptor: [Cardiomyopathies] explode all trees	2064
#2	MeSH descriptor: [Biomarkers] explode all trees	22,124
#3	MeSH descriptor: [Echocardiography] explode all trees	4289
#4	MeSH descriptor: [Peripartum Period] explode all trees	18
#5	MeSH descriptor: [Prognosis] explode all trees	165,063
#6	MeSH descriptor: [Pregnancy] explode all trees	23,920
#7	#1 AND #6	11

Science Direct

An advanced search strategy was utilized to include keywords and to also include at least the words "peripartum cardiomyopathy" in the title, abstract, or keywords. A filter was then used to include only the articles published in the last 15 years.

Table 6 shows our full strategy for Science Direct.

**Table 6:** Science direct search strategy (last search January 31, 2022)

Find articles with these terms	Year	Title, abstract, keywords	Open access articles only
Peripartum cardiomyopathy echocardiography biomarkers prognosis	2007-2022	Peripartum cardiomyopathy	Six

Google Scholar

An advanced strategy was utilized to include our keywords and to include at least one of the words "peripartum cardiomyopathy." A filter was then applied to include only the articles published in the last 15 years. The above search strategy revealed 5130 results. As sorted by relevance, the first 140 articles were then extracted.

Table 7 shows our full strategy on Google Scholar.

**Table 7:** Google Scholar search strategy (last search January 31, 2022)

Keywords	Year	Initial result	Articles extracted
Echocardiography biomarkers prognosis peripartum cardiomyopathy	2007–2022	5130	140
